# Neoadjuvant Treatment in Locally Advanced Pancreatic Cancer (LAPC) Patients with FOLFIRINOX or Gemcitabine NabPaclitaxel: A Single-Center Experience and a Literature Review

**DOI:** 10.3390/cancers11070981

**Published:** 2019-07-13

**Authors:** Fabiana Napolitano, Luigi Formisano, Alessandro Giardino, Roberto Girelli, Alberto Servetto, Antonio Santaniello, Francesca Foschini, Roberta Marciano, Eleonora Mozzillo, Anna Chiara Carratù, Priscilla Cascetta, Pietro De Placido, Sabino De Placido, Roberto Bianco

**Affiliations:** 1 Department of Clinical Medicine and Surgery, University of Naples “Federico II”, 80131 Naples, Italy; 2 Pancreatic Surgery Unit, Pederzoli Hospital, Peschiera del Garda, 37019 Verona, Italy

**Keywords:** LAPC, locally advanced pancreatic cancer, FOLFIRINOX, Gemcitabine Nab-Paclitaxel, neoadjuvant chemotherapy, NACT, conversion therapy

## Abstract

The optimal therapeutic strategy for locally advanced pancreatic cancer patients (LAPC) has not yet been established. Our aim is to evaluate how surgery after neoadjuvant treatment with either FOLFIRINOX (FFN) or Gemcitabine-NabPaclitaxel (GemNab) affects the clinical outcome in these patients. LAPC patients treated at our institution were retrospectively analysed to reach this goal. The group characteristics were similar: 35 patients were treated with the FOLFIRINOX regimen and 21 patients with Gemcitabine Nab-Paclitaxel. The number of patients undergoing surgery was 14 in the FFN group (40%) and six in the GemNab group (28.6%). The median Disease-Free Survival (DFS) was 77.10 weeks in the FFN group and 58.65 weeks in the Gem Nab group (*p* = 0.625), while the median PFS in the unresected group was 49.4 weeks in the FFN group and 30.9 in the GemNab group (*p* = 0.0029, 95% CI 0.138–0.862, HR 0.345). The overall survival (OS) in the resected population needs a longer follow up to be completely assessed, while the median overall survival (mOS) in the FFN group was 72.10 weeks and 53.30 weeks for the GemNab group (*p* = 0.06) in the unresected population. Surgery is a valuable option for LAPC patients and it is able to induce a relevant survival advantage. FOLFIRINOX and Gem-NabPaclitaxel should be offered as first options to pancreatic cancer patients in the locally advanced setting.

## 1. Introduction

Pancreatic Ductal Adenocarcinoma (PDAC) is an aggressive disease, being the fourth leading cause of cancer mortality, which is expected to achieve second place by 2030 [1]. Combined five-year survival for all stages is 9%, which ranged from 31–34% for radically resected patients to 3% for metastatic cases [2,3]. At time of diagnosis, more than half of the patients are in the advanced stage, with a palliative treatment being offered, while around 10–20% can undergo an upfront tumour resection [4]. Indeed, surgery with radical intent is the only treatment that is potentially able to offer a long-term survival. The remaining 25–35% of patients with pancreatic cancer have a borderline resectable (BR) or locally advanced (LA) disease (PC) [5]. These two definitions have been introduced in the last decade, but there is no consensus regarding how to correctly define the two entities [6]. 

Borderline resectable pancreatic cancers are tumours that could be a candidate for surgery after downsizing with neoadjuvant chemotherapy (NACT), while patients with locally advanced diseases have low chances of obtaining a margin-free resection and are offered palliative treatment as first option. The main cause of initial unresectability is blood vessels involvement [7]. 

FOLFIRINOX (FFN) [8,9] and Gemcitabine-NabPaclitaxel (GemNab) [10] have been introduced in the treatment of locally advanced pancreatic cancer patients (LAPC) after demonstrating their value in the metastatic setting. These schemes, at the cost of increased toxicity, are able to achieve longer overall survival (OS), longer progression free survival (PFS), and higher response rates (RR) when compared to Gemcitabine alone, even though randomized clinical trials in the LAPC setting have never been performed. 

The treatment of LAPC patients has been enriched with radiotherapy (RT) concurrent to chemotherapy after induction chemotherapy. Data from clinical trials with concurrent chemoradiotherapy (CHRT) treatments reported improved survival outcomes [11,12,13], interesting data came in terms of resection rates and clinical outcomes from retrospective analysis of patients undergoing surgery after neoadjuvant treatment while using chemoradiation [14], although this option is not evaluated in the current study. 

The therapeutic strategy to adopt in LAPC patients is controversial and a definitive answer is still missing. In such context, we conducted a monocentric retrospective study, as well as a systematic review of the literature to evaluate the role of FFN and GemNab in the treatment of LAPC, as part of a neoadjuvant strategy, where chemotherapy is used as induction treatment for definitive surgery.

## 2. Results

### 2.1. Patient Cohort

#### 2.1.1. Characteristics of the Patients

**Figure 1:**
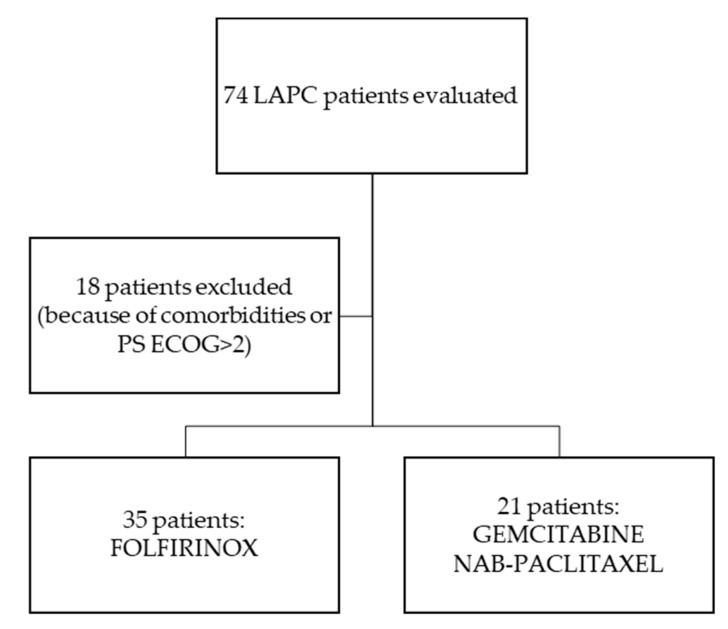
Flow chart in patients’ selection for analysis.

From October 2014 to May 2019, a total of 74 patients were evaluated at the Oncology Unit of Federico II University Hospital in Naples, Italy, with diagnosis of LAPC, as per the National Comprehensive Cancer Network (NCCN) Guidelines [15]. Eighteen patients because of comorbidities or PS ECOG>2 started treatment with a single agent chemotherapy or were offered Best Supportive Care (BSC) alone. Thirty-five patients started the FOLFIRINOX regimen and 21 Gemcitabine-NabPaclitaxel (Figure 1); see “Treatments” for the regimens’ specifics.

Table 1 shoes the demographic and disease characteristics of the patients at baseline. The populations were similar in the two treatment groups, except in the FFN treatment group age (59.2 years versus 65.4; *p* = 0.042) and PS ECOG (*p* = 0.007) were both statistically significantly lower.

#### 2.1.2. Treatments

The patients analysed were treated with neoadjuvant chemotherapy treatment (NACT), with either FFN (Oxaliplatin 85 mg/mq + Calcium Levofolinate 200 mg/mq + Irinotecan 180 mg/mq + 5fluorouracile 400 mg/mq bolum + 5fluorouracile 2400 mg/mq continuous infusion 46 h g1 q14) or GemNab (Gemcitabine 1000 mg/mq + NabPaclitaxel 125 mg/mq g1,8,15 q28).

The median number of cycles before surgery was 11.5 (range 6–15) in the FFN group and five (range 3–6) in the GemNab group. Patients not undergoing surgery treatment had a median number of 10 cycles of therapy (range 2–12) in the FFN group and six (range 3–8) in the GemNab group, before interrupting for progression, death, or intolerability. The median duration of treatment was 26.43 weeks (range 10.00–36.86) for patients undergoing surgery in the FFN group and 23.64 weeks (range 2.00–29.00) in the group not undergoing surgery (*p* = 0.6794); in the GemNab group, 18.9 weeks (range 11.43–26.00) and 12.71 weeks (range 9.86–34.43), respectively (*p* = 0.4562). Patients that were still on treatment at the time of analysis were excluded. In the FFN group, 51.43% of patients had delays or dose reduction (median reduced dose 80%, range 70–100%) and 48% in the GemNab (median reduced dose 80%, range 80–100%). The percentage of patients after surgery who underwent local radiotherapy was 53.8% in the FFN group and 40% in the GemNab group. The percentage of patients that underwent local radiotherapy treatment before surgery, as per surgeon request, was 20%, with two patients in each group.

#### 2.1.3. Efficacy

**Figure 2:**
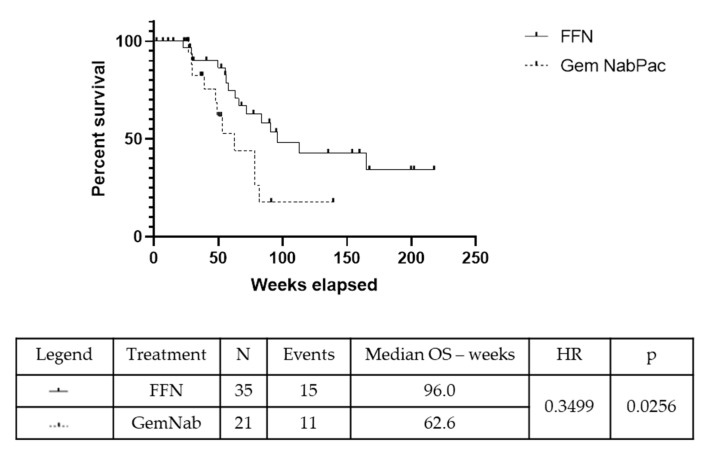
Kaplan-Meyer curve for OS in the overall population (OS pop) of patients treated with either FFN or GemNab. The median Overall Survival (mOS) in the overall population was 96 weeks in the FFN group and 62.6 weeks in the GemNab group (*p* = 0.026, 95% CI 0.14–0.88; HR = 0.35).

The median duration of follow up was 12.8 months. The number of patients undergoing surgery was 14/35 with FFN (40%) and 6/21 with GemNab (28.6%) (*p* = 0.565). The R0 resection rate was 10/14 (71.43%) in the FFN group and 5/6 (83.3%) in GemNab group (*p* = 0.87). The remaining five patients obtained R1 resections (four in the FFN group, one in the GemNab); no R2 resections were registered. The value of the Carbohydrate Antigen 19.9 (CA19.9) after neoadjuvant therapy was evaluated to find correlation with the status of margins at resection. In the FFN group: one patient had an increase in CA19.9 value of 125%, obtaining a R0 resection; one patient had a decrease in the CA19.9 value of less than 50%, with an R1 resection; the remaining 12 had a decrease in the CA19.9 value 50–100% of the baseline value, with three R1 resections and nine R0s. In the group of patients resected after GemNab: one patient had an increase of almost 300% of the baseline value of CA19.9 and underwent resection, with positive margins; one patient had a reduction of 0–50% of the CA19.9 value and obtained a R0 resection; two patients had a CA19.9 reduction of 50–100% with both obtaining R0 resections. We have not obtained information regarding the CA19.9 after NACT before surgery in two patients. Statistical analysis was not performed because of the small sizes of the subgroups. The median Overall Survival (mOS) in the overall population was 96 weeks in the FFN group and 62.6 weeks in the GemNab group (*p* = 0.026, 95% CI 0.14–0.88; HR = 0.35) (Figure 2). 

**Figure 3:**
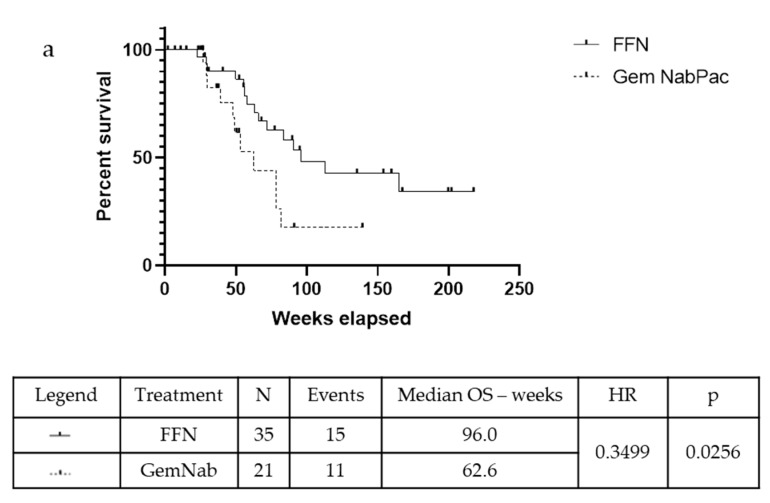

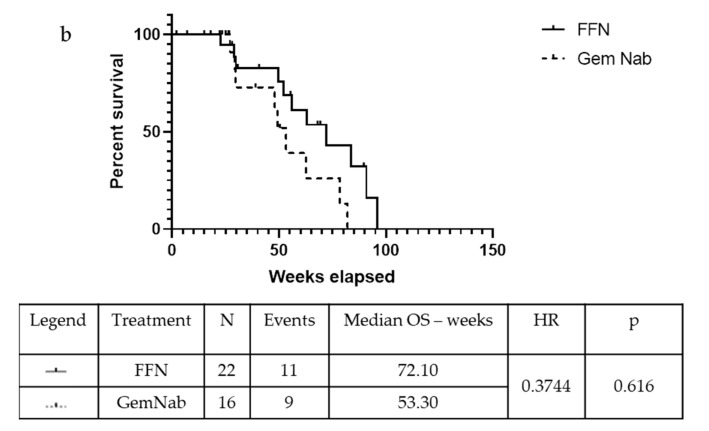
Kaplan-Meyer curve for OS (a) and PFS (b) of patients not undergoing surgery in FFN and GemNab groups. (**a**) Kaplan-Meyer curve for OS of patients not undergoing surgery in FFN and GemNab groups. In the unresected population, the mOS was 72.10 weeks in the FFN group and 53.30 weeks in the patients treated with GemNab: although not statistically significant, there is a positive tendency in favour of FFN treatment (*p* = 0.06, 95% C.I. 0.13–1.05; HR = 0.37). (**b**) Kaplan-Meyer curve for PFS of patients not undergoing surgery in FFN and GemNab groups. The median Progression Free Survival (mPFS) was 49.40 weeks in the FFN unresected group and 30.90 weeks in the GemNab unresected group (*p* = 0.0029, 95% C.I. 0.131–0.863; HR = 0.35).

In the unresected population, the mOS was 72.10 weeks in the FFN group and 53.30 weeks in the patients that were treated with GemNab: there is a positive tendency in favour of FFN treatment, although not statistically significant (*p* = 0.06, 95% CI 0.13–1.05; HR = 0.37) (Figure 3a). The median Progression Free Survival (mPFS) was 49.40 weeks in the FFN group and 30.90 weeks in the GemNab group (*p* = 0.0029, 95% CI 0.131–0.863; HR = 0.35) (Figure 3b).

**Figure 4:**
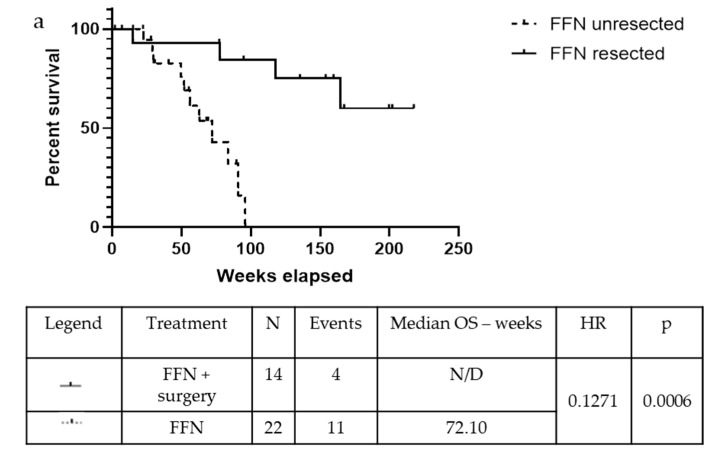

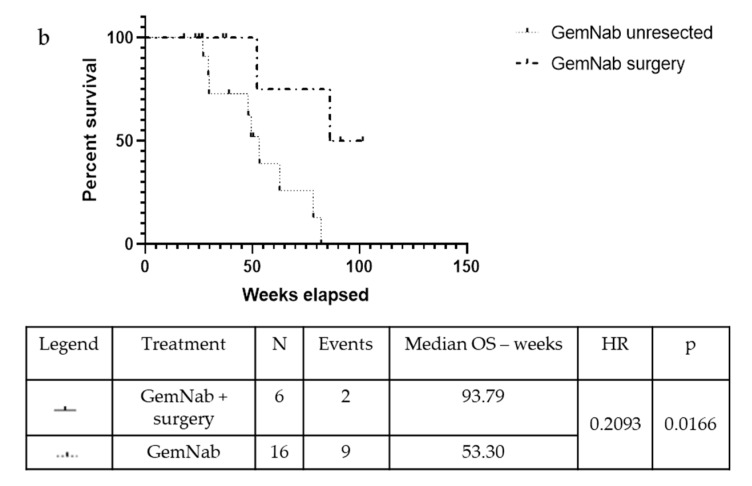
Kaplan-Meyer curve for OS confronting patients who underwent surgery versus unresected patients, after treatment with neoadjuvant FFN (a) or GemNab (b). (**a**) The median Overall Survival (mOS) was not reached in the FFN resected group and 72.10 weeks in the FFN unresected group (*p* = 0.0006, 95% C.I. 0.03935–0.4105; HR = 0.13). (**b**) The median Overall Survival (mOS) was 93.79 weeks in the GemNab resected group and 53.30 weeks in the GemNab unresected group (*p* = 0.0166, 95% CI 0.05822–0.7523; HR = 0.21).

The median Disease-Free Survival (DFS) was 77.10 weeks in the FFN group and 58.65 weeks in the GemNab group in the population undergoing surgery (*p* = 0.625). Meanwhile, the mOS is yet to be reached in the FFN group and it is 93.79 weeks in the GemNab patients; moreover, analysing the difference in overall survival between unresected and resected patients is statistically significant, both in the FFN (*p* = 0.0006; Figure 4a) and in the GemNab group (*p* = 0.0166; Figure 4b). A longer follow up is still needed in the population undergoing surgery. 

**Figure 5:**
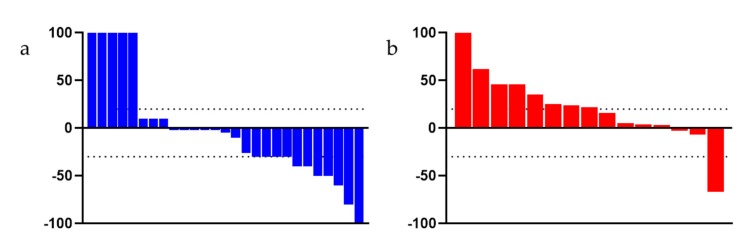
Waterfall Plot evaluating the objective responses of patients, (**a**) treated with FFN and (**b**) treated with GemNab. The dotted bar indicates +20% and −30%, range for stable disease, as per RECIST criteria.

In the overall population, the OS rate at 12 months and 18 months was 71.42% and 45.71% in the FFN group and 52.38% and 28.57% in the GemNab group, respectively. At tumour relapse (disease relapse or progression disease), 100% of patients in the FFN group and 90.9% in the Gem Nab started a chemotherapy treatment. Staging reassessment after surgery showed a Disease Control Rate (DCR) of 68.57% and an Overall Response Rate (ORR) of 48.57% in the FFN group, with 17 partial responses, seven stable diseases (of which two underwent surgery), seven patients had progression disease or died, and the assessment was unavailable in four patients. In the GemNab group, DCR was 57.14% and ORR was 33.33%, with partial response in seven patients, five stable diseases (of which one patient underwent surgery); seven patients progressed or died and the assessment was not yet available for two patients. Both DCR and ORR are not statistically significant (*p* = 0.57 and *p* = 0.40, respectively). Figure 5 shows the objective responses. 

At the time of analysis, a cancer-related event (local or distant progression) or death occurred in 24 patients (68.57%) in the FFN group and in 11 patients (52.38%) in the GemNab group. A total of 29 patients relapsed (51.79%): 18 in the FFN group, of which seven patients presented a local recurrence, while 11 developed distant metastases. In the GemNab group, 11 patients relapsed: five locally and six at distant sites (Table 2). Twenty patients were still alive at study completion, 14 in the FFN group and six in the GemNab.

#### 2.1.4. Prognostic Factors

The patients’ characteristics were used to evaluate the prognostic factors influencing the survival outcomes. Although the numbers of patients in each subgroup is small, we observed a positive influence on OS (Figure S1a) and PFS (Figure S1b) of age less than 65 years, ECOG 0 and negative nodes involvement in the FOLFIRINOX treatment group. A longer follow up is needed to further evaluate these factors.

#### 2.1.5. Adverse Events

Adverse events of grade 3 or 4 were reported in 37.14% of patients in the FFN group and in 23.81% in the GemNab group (Table 3). The more frequent grade 3 or 4 adverse events were neutropenia in the FFN group and diarrhoea in the GemNab group. The occurrence of Neutropenia G1–2 was 31.43% and G3–4 was 28.57% in the FFN group, while it appeared as G1–2 in the 28.57% and 4.76% as G3–4 in the GemNab group. Significant differences in main toxicities were not seen between the two treatment groups.

### 2.2. Review of the Literature

Multiagent chemotherapy treatment schedules have only recently been offered to locally advanced pancreatic cancer patients. Hereby, we reviewed the published data from retrospective analysis and randomized clinical trials (RCTs), on the use of FFN or GemNab in LAPC patients.

#### 2.2.1. FOLFIRINOX

No results from randomized clinical trials evaluating the role of FFN in locally advanced patients are available yet. However, several case series were also published on the use of FFN in the LAPC setting after the results of the PRODIGE4/ACCORD11 trial [9], where in the metastatic setting a prolonged survival was obtained (11.1 months with FFN vs. 6.8 months with gemcitabine alone), accompanied by an overall response rate (ORR) of 32% (vs. 9%, *p* < 0.001). 

Most of the manuscripts evaluated LAPC patients, together with metastatic or borderline resectable patients, so the survival results and/or resection rates of the subgroups are not often known [16,17,18]. In 2013, Petrelli and colleagues published a meta-analysis [19] evaluating 13 studies, in which patients (253) with borderline resectable pancreatic cancer (BRPC) or LAPC were treated with neoadjuvant FFN: the pooled rate of LAPC resection was 26.1% (95% C.I. 18.2–35.9%) and the rate of R0 resections was 22.5% (95% C.I., 13.3–35.4%) [20]. A more recent systematic review was performed by Rombouts and colleagues [14] that evaluated 14 studies, including 365 patients exclusively with a LAPC diagnosis, of which 57% were treated with radiotherapy (alone or with concurrent chemotherapy) after neoadjuvant FFN: 103 patients underwent resection (28%), of which 72 were R0 (77%). The authors also solely analysed the outcomes of FFN treatment: the pooled resection rate was only 12% (29/242 patients), with 14 R0 resections (out of the 20 available for assessment). However, the authors could not perform a meta-analysis of the OS, because of the heterogeneity between the studies and the lack of individual patient data. Eventually, a complete analysis of the survival benefit that was gained after treatment of LAPC patients with FOLFIRINOX was published by Suker [21]. The analysis included thirteen non-randomized studies, with 355 LAPC patients, assessing survival benefit, resection rate, and tolerability. The mOS was 24.2 months (95% CI 21.7–26.8) and the median PFS was 15.0 months (95% CI 13.7–16.3). Of the 325 patients that were evaluated for surgery, 91 were eligible (pooled proportion 25.9%) and R0 resections were obtained in 60 (74%) of the 81 assessable patients. The percentage of patients that were treated with radiotherapy or chemioradiotherapy varied from 31% to 100% across studies: there was no association between the proportion of patients undergoing these treatments and OS. The Sadot and colleagues study [22] was included in the Suker analysis: one of the largest series assessing FFN in 101 LAPC patients, all staged as T4 any N M0. In the analysed population, almost one-third underwent tumour resection and almost 50% was offered chemoradiotherapy as alternative treatment. The R0 resections were obtained in 55% of the patients. Other studies evaluating the efficacy of chemoradiation demonstrated a low R0 resection rate [13,23]. The median OS was similar in recently published retrospective analyses, being more than two years in patients undergoing surgery after neoadjuvant treatment, while mDFS could vary from 9.0 months to 48 months [24,25,26]. Opposing these results, the survival data were weaker in the Japanese national observational study [27]. 

Several studies demonstrated how FFN as NACT before surgery could be a manageable treatment option with high rate of R0 resection with [28] or without radiotherapy [29,30]. Opposite to those, the studies by Barenboim [31] and Suker [32] registered a low resection rate, which do not support the use of FFN to convert primary non-resectable cancer to resectable. Therefore, the resection rates can profoundly vary, from a 23.4% in the Lee [33] analysis with 64 patients evaluated, to a 44% in the Blazer [34] study where the patients’ population was limited to 25 patients. However, in the biggest series available, such as those by Hackert and colleagues [35], the RR is extremely high: of 575 patients retrospectively evaluated, 125 were offered FFN, of which 61% were finally resected. Finally, the results from a phase II trial (IMPALA) [36] showed that, of 61 patients treated with FFN, 36 had an exploratory laparotomy and 14 (39%) underwent tumour resection. In this trial, the patients not suitable for surgery were offered as alternative irreversible electroporation (IRE), but the difference in mOS is wide: 34 months for the resected group and 16 months in the patients undergoing IRE. 

Toxicities that were related to FFN treatment have always been an issue: grade 3 or 4 most frequent adverse events are neutropenia, febrile neutropenia, thrombocytopenia, diarrhoea, fatigue, nausea, and G2 sensory neuropathy [9]. To reduce their frequency, several authors applied the modified FFN scheme (most of times without the 5FU bolus, sometimes even with dose reduction of the other chemoterapeutics drugs). Among these, Nanda and colleagues [37] retrospectively analysed 29 LAPC patients that were treated with modified (mFFN), followed by chemoradiation (with either concomitant gemcitabine or capecitabine) and obtained a 41.9% resection rate (12 patients), of which 10 patients had a margin-free resection. In this study, the toxicities were reduced in the modified protocol. Stein and colleagues obtained similar results [38], which treated 31 patients and obtained a 41.9% RR, with a mPFS of 17.8 months and a mOS 26.6 months. However, using mFFN Lakatos [39] obtained only a 6.3% resection rate (in 32 patients) and it did not reported toxicity reduction, as expected. Regarding the use of the mFFN scheme, Khushman and colleagues [40] demonstrated that the dose intensity and duration were associated with increased survival, arguing against dose attenuated versions of this regimen. Opposite to this study, several studies recently demonstrated that the reduced doses of the FFN scheme did not have an impact on the survival [41,42].

Table 4 includes the abovementioned studies. 

The results and the experience accumulated through the years regarding the use of FFN as NACT are very controversial. A phase III study of mFFN with or without RT in patients with LAPC is now ongoing to try to clarify the potential of this therapy option, with mPFS as the primary endpoint [NCT01926197].

#### 2.2.2. Gemcitabine-NabPaclitaxel

In 2013, Von Hoff published the results from the MPACT trial [10], where GemNab combination therapy offered a longer OS in metastatic PC patients (8.5 months with GemNab versus 6.7 months with gemcitabine alone). Recently, this regimen has started to be used in the neoadjuvant setting. 39 patients were treated with GemNab in a study from Montes and colleagues [43] (nine LAPC and 30 mPC): ORR and DCR were 23% and 81%, respectively, and mPFS was nine months, while mOS was 15 months. Peterson and colleagues [44] in 2018 published a retrospective analysis of 32 patients that were treated with GemNab as NACT, of which 10 were LAPC: two obtained a R0 resection, with a mOS of 10.4 months. A phase I study was performed in Japan [45], where only LAPC patients were enrolled: patients were offered GemNab with the weekly administration of 800 mg/m^2^ of gemcitabine and 100 mg/m^2^ of nab-paclitaxel with concurrent RT (50.4 Gy). Six of the 12 patients enrolled proceeded to surgery, so confirmatory prospective trials are awaited in order to further prove the efficacy of such an approach. A similar phase I study was performed by Shabason and colleagues [46], where two dosing regimens were confronted in a 3 + 3 dose escalation strategy: five LAPC patients were treated with either nab-paclitaxel 100 mg/m^2^ or 125 mg/mq, of which two underwent surgical R0 resection. The trial resulted in the confirmed safety and tolerability of the full dose treatment. A small retrospective analysis was published in 2017 [47], where seven LAPC patients were treated with GemNab, of which two underwent a R0 resection, with a median survival for all the patients of 13.3 months. 

At ASCO GI 2018, the results from the interim analysis of the phase II trial LAPACT [48] have been presented: this was an international, multicentre, open label, single-arm study that used Gemcitabine-NabPaclitaxel as induction therapy in patients with unresectable locally advanced PDAC. The patients were re-assessed after a maximum of six cycles: the investigator could choose among several options in the absence of disease progression or unacceptable toxicity (continuing the same chemotherapy, offer chemo-radiation, or evaluate surgical resection). Primary endpoint was the time to treatment failure. At the time of analysis, 16 patients had undergone surgery (15%), of which seven patients were R0, 17 patients (16%) had received chemoradiation, 13 (12%) continued GemNab treatment [48]. The results are not available yet. A similar study started in 2014 by the Italian Group’s (GISCAD), the GAP trial, when compared Gemcitabine alone versus GemNab for LAPC patients [NCT02043730]; final results have yet to be published. Table 5 includes all of the studies analysed with published data or abstract.

#### 2.2.3. Studies Involving both FFN and GemNab

In 2019, Rangelova and colleagues published the results of their retrospective analysis [41], where 21 patients started the GemNab treatment, the largest series so far, together with our study. However, the impact on the survival has not been evaluated with GemNab, but only in the FFN group, which counted 54 patients. Interestingly, the authors found no differences in survival between BRPC and LAPC patients and decided to evaluate them together in the statistical analysis (OS BRPC 15.0months versus LAPC 14.5months, *p* = 0.4; OS BRPC resected patients 31.9 months versus LAPC resected patients 21.8 months, *p* = 0.7). Another retrospective analysis by Gemenetzis and colleagues [49] evaluated 415 LAPC patients: among them, 158 patients were offered FFN and 16 GemNab. Fifty-three patients in the FFN group were resected, while the resectability rate was not available in the GemNab group. The mOS in the FFN resected subgroup was 35.3 months and 16.2 months for the unresected population. Patients not surgically resectable, which had been treated with radiotherapy after induction chemotherapy, obtained an OS benefit (20 months versus 14 months). The relapse free survival was 11.3 months. The studies evaluating both treatments, FFN and GemNab, are included in Table 6. In addition, several studies are ongoing comparing the two regimens as NACT, or as induction therapy to other treatment (see Table S1).

## 3. Discussion

For decades, LAPC patients have been offered first line palliative treatments that are used in the metastatic setting, to obtain disease control, preservation of quality of life, and if possible, improvement in the OS. In these cases, OS has always been in the range of few months: from the study of Burris in 1997 [50] demonstrating a survival benefit in LAPC and mPC patients of gemcitabine versus 5fluorouracil alone, 5.6 vs. 4.4 months, to later studies evaluating gemcitabine as first line treatment in LAPC, demonstrating a median OS of 6–13 months [11].

In 2005, LAPC patients, together with metastatic patients, were offered the opportunity to be treated with a polichemotherapy scheme: FFN demonstrated a survival benefit in both groups of patients [8]. Although locally advanced patients have been removed in the subsequent phase III trial, after the results that were obtained in the PRODIGE4/ACCORD11 [9] were published, FFN started to be used, even in the locally advanced setting. After several experiences were published, Suker and colleagues [21] published an interesting patient-level meta-analysis, including the data from retrospective studies as well as randomized control trials, evaluating the role of FFN in LAPC patients. In the Suker’s pooled analysis, the survival benefit has been demonstrated: mOS, ranging from 10.0 to 32.7 months, was 24.2 months [21]. This survival is much longer than that observed in metastatic patients and it is more similar to that reported in resected patients. 

In addition to that, to control local progression and achieve an impact on the survival outcome, radiotherapy treatments (SBRT, IMRT, etc.,) have been introduced for LAPC patients. Ultimately, surgery has entered the arena. This is the proof of the paradigm shift that happened through the years: locally advanced pancreatic cancer patients have been offered more than just palliative treatments. 

Even though the surgery is the only option that is known to offer a concrete survival benefit to pancreatic cancer patients, doubts seem to be still present in the surgeons’ opinion. Nevertheless, it has been long demonstrated in several studies that the high mortality rates that were related to the pancreatic resection procedures [51] could be reduced to <5% if the surgery is performed in experienced centers [52]. The burden of the surgical complication is not overwhelming anymore and surgery can be safely performed in experts’ hands [53].

Moreover, a special mention should be done regarding radiotherapy treatment, although this study did not focus on its role. Nowadays, there are still contrasting evidences regarding studies that suggest a survival benefit in patients that did not undergo surgery because of high risk of a R1 margin resection, where OS could be compared to definitive chemoradiation without surgery [54,55,56], against studies that, conversely, display a considerably longer OS when surgery is performed anyway [57]. 

Nevertheless, a multidisciplinary teamwork is desirable in LAPC patients, in order to offer the best therapeutic options, including induction chemotherapy followed by surgery. Moreover, through a more efficient neoadjuvant strategy, the role of surgery should be carefully assessed in LAPC patients. In addition, some recently published studies suggest that CT scan is often not able to precisely predict the downstaging in primary pancreatic lesions upon neoadjuvant chemotherapy [18], warranting a surgical exploration after NACT for all the patients [58].

In our retrospective analysis, we demonstrated that LAPC patients can be addressed to surgery to obtain DFS and OS benefits. After a median follow up of more than 12 months, the mOS was not reached in the FFN resected group and it was around 93.79 weeks in the GemNab resected group when compared to 72.10 weeks and 53.3 weeks in the unresected groups, respectively (*p* = 0.0006 in the FFN group and *p* = 0.0166 in the GemNab group). Moreover, patients that could not undergo surgery still gained an increase in PFS after the neoadjuvant therapy (49.4 weeks versus 30.9, *p* = 0.0029). The benefit of using a multidisciplinary approach and comprehensive surgery evaluation is clearly valuable when comparing the mPFS and the mOS values of these patients with historical data. Data on the OS in the resected population are not complete yet; moreover, a long-term follow-up is necessary for evaluating the prognostic effect of the surgical strategy.

Several questions are still unanswered, including (a) which is the best therapeutic options as NACT, (b) which is the timing for the surgery, and (c) how many cycles of chemotherapy are needed before abandoning the “resection goal” and go for definitive RT.

Some questions will be addressed by ongoing clinical trials, including those comparing FFN and GemNab as neoadjuvant strategies, with or without RT (www.clinicaltrials.gov) (Table S1). 

Moreover, some of these studies aim to find predictive biomarkers to stratify patients, in order to offer the best fitting therapeutic strategy each time. First of all, almost 10% of the PDAC patients demonstrated a familial predisposition by BRCA, and related genes, mutations [59]. In the presence of BRCA mutations, a platinum based chemotherapy seems to be the best choice [60], although data in the LAPC setting are still lacking and more studies are warranted. In addition, poly (ADP-ribose) polymerase (PARP) inhibitors have demonstrated great anti-tumour activity in BRCA mutated patients and they have recently been evaluated as maintenance therapy in PDAC in the first line setting after a platinum-based chemotherapy [61]. Their role in LAPC patients could be interesting. Currently, trials with concurrent or sequential platinum plus PARP inhibitor are under evaluation (www.clinicaltrials.gov). In addition, genetic markers, such as single nucleotide polymorphism (SNP), circulating tumour DNA (ctDNA), long non-coding RNAs (lnRNAs), and immunologic markers, such as interleukin, TGFβ, CD40L, etc., have been evaluated as predictive factors: unfortunately, none of the studied circulating biomarkers met the criteria for “predictive biomarkers” [62]. Nevertheless, these studies provided background for further research. Studies that particularly evaluate biomarkers predicting the response to FFN or GemNab are very rare, even though these schemes are currently the best therapeutic options for PDAC patients. Recently, the role of the soluble form of CD40L (sCD40L), a molecule that belongs to the tumour necrosis factor family, has been investigated in patients that were treated with FFN or GemNab as first line treatment [63] and its higher levels at the end of the treatment seem to be related to unresponsiveness to the chemotherapy treatment. Soluble CD40L has been previously found to be involved in the regulation of the immune reaction and fibrosis in PDAC, and perhaps it could hold potential as a prognostic marker [64]. Moreover, the role of sCD40L in modulating the tumour microenvironment (TME) through the regulation of the immune reaction and fibrosis in PDAC have been investigated [65] and is currently being evaluated in an ongoing phase I trial [66]. Pancreatic cancer TME consists of a stroma enriched in proteins that are produced by cancer associated fibroblasts (CAFs), which develop from bone marrow-derived mesenchymal stem cells (MSCs), pancreatic stellate cells (PSCs), and quiescent resident fibroblasts through multiple pathways [67]. These cells play an important role in tumour proliferation, progression, and invasion [67]. Recently, the role of CAFs and mast cells in affecting the sensitivity of PDAC cells to GemNab has been investigated [68]. The authors utilized cellular models to demonstrate that, while CAFs seem to not affect the combination effectiveness, mast cells crosstalk with PDAC cells strongly reduced GemNab anti-tumour activity through TGFβ signalling [68]. Even though a solution has yet to be found, biomarkers predicting the responses to chemotherapy are fundamental for patient stratification, to select the most appropriate pharmacological agent to finally reach the personalization of the treatment. In our study, none of the analysed factors (site of the lesion, CA19.9 baseline or changes pre- and post-treatment, neutrophils to lymphocytes ratio, etc.) have been significantly associated with survival outcomes.

Our study has some strengths as well as limitations. Firstly, it is the first retrospective analysis, where only LAPC patients have been evaluated, and then excluding resectable or borderline resectable or metastatic diseases. Second, the mOS in the unresected population is a significant demonstration of the importance of a complete therapeutic strategy that should be offered to LAPC patients, confirming the great conceptual distance with the metastatic setting. Finally, the mDFS of more than 17 months and mOS yet to be reached demonstrates that LAPC patients should be addressed to surgery, whenever feasible. The limitations in this study are the small number of patients evaluated, the longer follow up that is needed to observe a clear survival benefit, and, even more, for the correct evaluation of prognostic factors, since the number of patients in the subgroups are small. Finally, there was a bias in offering the treatments to the patients, with FFN more frequently being chosen for younger patients with better PS ECOG.

## 4. Materials and Methods 

### 4.1. Patients Selection

Patients, at least 18 years of age, which came to our Department of Oncology, at “Federico II” University of Naples with cytologically or histologically confirmed pancreatic ductal adenocarcinoma (PDAC), who had been evaluated as LAPC, while using NCCN guidelines as reference, with no evidence of metastatic disease, malignant ascites, or pleural effusion, were retrospectively evaluated and included in the analysis. 

The information that was collected at baseline included the patients’ Performance Status assessed following the ECOG scale (PS ECOG), CA19.9 value, haematological function, lesion site (head versus body/tail), weight, and all of the mentioned value were assessed again at the end of the neoadjuvant treatment. 

Patients that underwent surgery had a stage re-assessment with CT scans. As per surgeon or oncologist request, some of the patients had local radiotherapy after neoadjuvant chemotherapy and before surgery. Some of the patients that did not undergo surgery had local radiotherapy and then were offered follow up. Information regarding adverse events, graded according to the National Cancer Institute Common Terminology Criteria for Adverse Events version 5.0, was collected.

### 4.2. Study Design

This monocentric retrospective study evaluated patients with LAPC who underwent neoadjuvant treatment with either FOLFIRINOX (Oxaliplatin 85 mg/mq + Calcium Levofolinate 200 mg/mq + Irinotecan 180 mg/mq + 5fluorouracile 400 mg/mq bolum + 5fluorouracile 2400 mg/mq continuous infusion 46 h g1 q14) or Gem-NabPaclitaxel (Gemcitabine 1000 mg/mq + NabPaclitaxel 125 mg/mq g1,8,15 q28), as the chemotherapy scheme. Treatment delays or reduction were registered, and they were related to the occurring of adverse events or because of patients’ decision.

### 4.3. Efficacy and Survival Outcomes

OS was calculated from the date of first administration of NACT until death from any cause. DFS was calculated from the date of surgery until the date of the first cancer-related event or death for any cause. PFS was calculated from the date of first administration of NACT, until the date of the disease progression or death for any cause. 

Consolidation therapy after NACT were recorded: chemoradiotherapy, or radiotherapy alone, surgery, quality of the resection. Local or metastatic relapse and death happening during the NACT or after were recorded. In the case of disease progression, data regarding the following treatments were recorded.

### 4.4. Statistical Analysis

We generated curves to describe time-to-event variables (OS and PFS), according the Kaplan Meier method. Survival curves were compared while using the long-rank test. Prism 7.04 software (GraphPad, San Diego, CA, USA) and R Studio (Integrated Development for R. RStudio, Inc., Boston, MA, USA) were used to perform statistical analysis and a graphical plotting of results. The results were considered to be significant when the *p*-value was less than 0.05. 

### 4.5. Ethics

The Federico II University of Naples Ethic Committee (ethic code: 159/19) approved the study protocol on 12 June 2019.

### 4.6. Literature Data

PubMed database was searched while using the terms “locally advanced pancreatic cancer”, “FOLFIRINOX”, “Gemcitabine Nab Paclitaxel”, and their combination. Retrospective series, prospective trials and randomized clinical trials accessible in full text or as abstract presented in congresses were considered in the analysis. Case reports were excluded.

## 5. Conclusions

Neoadjuvant chemotherapy treatment with either FFN or GemNab should be offered to LAPC patients. The response to the treatment is better evaluated trough surgical exploration, thus the CT scans cannot always precisely identify the clinical response. Finally, the magnitude of survival benefit in this setting of patients suggests that LAPC patients should always be evaluated for surgery.

## Figures and Tables

**Table 1:** Characteristics of the patients at the baseline. * indicates statistically significant *p* values.

	FOLFIRINOX (*n* = 35)	Gemcitabine NabPaclitaxel (*n* = 21)	*p*
Median Age years (range)	59.2 (42–70)	65.4 (44–79)	0.042 *
Male/Female	17/18	13/8	*0.12*
Site (n):			
Head Body/Tail	20 15	16 5	*0.46*
PS ECOG:			
0 1	30 5	17 9	0.007 *
Median CA19.9 UI/mL at diagnosis (range)	841.95 (1–10694)	2125.58 (0.8–18801)	*0.20*
T classification:			
T4 T3	27 8	19 2	*0.96*
N classification:			
N0 N+	14 21	7 14	*0.34*

**Table 2:** Recurrence pattern following resection in 29 LAPC patients.

	FOLFIRINOX (n = 18)	Gemcitabine NabPaclitaxel (n = 11)
Local Relapse Only	7	5
Distant Relapse:	11	6
Carcinosis	4	1
Lung	2	1
Liver	1	1
≥2 sites	4	3
Total Number of relapse—*n* (%)	29 (51.79%)	

**Table 3:** Toxicities of the treatments.

	FOLFIRINOX *n* = 35 (%)		Gemcitabine NabPaclitaxel *n* = 21 (%)	
	G1/2	G3/4	G1/2	G3/4
Anemia	20 (57.14)	0 (0)	17 (80.95)	1 (4.76)
Neutropenia	11 (31.43)	10 (28.57)	6 (28.57)	1 (4.76)
Decreased Platelet Count	15 (42.86)	1 (2.86)	6 (28.57)	1 (4.76)
Increased Transaminases	13 (37.14)	1 (2.86)	13 (61.90)	1 (4.76)
Oral Mucositis	6 (17.14)	0 (0)	2 (9.52)	0 (0)
Diarrhoea	16 (45.71)	2 (5.71)	8 (38.10)	2 (9.52)
Nausea	19 (54.29)	1 (2.86)	10 (47.62)	0 (0)
Vomiting	9 (25.71)	0 (0)	7 (33.33)	0 (0)
Fatigue	21 (60)	0 (0)	17 (80.95)	0 (0)
Parestesia	24 (68.57)	0 (0)	5 (23.81)	1 (4.76)
Total	91.12%	8.88%	92.86%	7.14%

**Table 4:** Studies evaluating FFN or mFFN included in the systematic review.

Author	Year	Treatment	N° of Patients Treated	Resection Rate (RR %)	R0 Resection (*n*/Total Resected Patients)	Reference
Barenboim	2018	FFN	30	3 (10%)	N/A	31
Blazer	2015	FFN	25	11 (44%)	10/11	34
Cavanna	2019	mFFN	13	N/A	N/A	42
Faris *	2013	FFN	22	5 (23%)	5/5	23
Ferrone *	2015	FFN	25	N/A	NA	18
Gunturu	2015	FFN	16	2 (12.5%)	N/A	16
Hackert	2016	FFN	125	76 (61%)	31/76	35
Hosein	2012	FFN	14	6 (43%)	5/6	17
Khushman	2015	FFN	40	N/A	6	40
Kourie	2019	FFN	14	14 (100%)	14/14	24
Lakatos	2017	mFFN	32	2 (6.3%)	2/2	39
Lee	2018	FFN	64	15 (23.4%)	11/15	33
Li	2019	FFN	41	14 (34.15%)	N/A	26
Mancini	2018	FFN	23	1 (4%)	1/1	13
Marchegiani *	2018	FFN	46	N/A	N/A	29
Matsumoto	2019	FFN	66	N/A	N/A	27
Nanda	2015	mFFN	29	12 (41.9%)	10/12	37
Nitsche	2015	FFN	14	4 (29%)	3/4	20
Petrelli °	2015	FFN	N/A	(26.1%)	(22.5%)	19
Pouypoudat	2019	FFN	38	13 (34.2%)	12/13	28
Rombouts ^#^	2016	FFN	365	103 (28%)	72/103	14
Sadot	2015	FFN	101	31 (31%)	16/31	22
Stein	2016	FFN	31	13 (41.9%)	13/13	38
Suker °	2016	FFN	355	91 (25.9%)	60/81	21
Wagner *	2017	FFN	13	13 (100%)	11/13	30
Yoo	2019	FFN	70	66 (94.29%)	N/A	25

* Only surgical resected case; # Systematic Review; ° Meta-Analysis; FFN: FOLFIRINOX; mFFN: modified FOLFIRINOX; N/A: Not Available.

**Table 5:** Studies evaluating GemNab included in the systematic review.

Author	Year	Treatment	N° of Patients Treated	Resection Rate (RR %)	R0 Resection (*n*/Total Resected Patients)	Reference
Yamada	2018	GemNab	12	6 (50%)	N/A	45
Montes	2017	GemNab	9	N/A	N/A	43
Peterson	2018	GemNab	10	N/A	N/A	44
Saito	2017	GemNab	7	2 (28,6%)	2/2	47
Shabason	2018	GemNab	5	2 (40%)	2/2	46
Hammel	2018	GemNab	107	16 (15%)	7/16	48

Gem Nab: Gemcitabine NabPaclitaxel; N/A: Not Available

**Table 6:** Studies both FFN and GemNab included in the systematic review.

Author	Year	Treatment	N° of Patients Treated	Resection Rate (RR %)	R0 Resection (*n*/Total Resected Patients)	Reference
Gemenetzis	2018	FFN	187	51 (63%)	N/A	49
		GemNab	16	N/A	N/A	
Rangelova	2019	FFN	54	N/A	N/A	41
		GemNab	21	N/A	N/A	

FFN: FOLFIRINOX; mFFN: modified FOLFIRINOX; Gem Nab: Gemcitabine NabPaclitaxel; N/A: Not Available.

## Supplementary Materials

The following are available online at https://www.mdpi.com/2072-6694/11/7/981/s1, Figure S1: Forrest Plot. Evaluating the characteristics of the patients and the tumour, possibly influencing the (a) OS and (b) PFS. Table S1. Active and/or ongoing clinical trials evaluating FOLFIRINOX and Gemcitabine NabPaclitaxel as neoadjuvant treatments.
