# Supplementary material for: Neoadjuvant Treatment in Locally Advanced Pancreatic Cancer (LAPC) Patients with FOLFIRINOX or Gemcitabine NabPaclitaxel: A Single-Center Experience and a Literature Review
Source: Cancers (Basel). 2019 Jul 13;11(7):981. doi: 10.3390/cancers11070981 (PMC6678351; doi:10.3390/cancers11070981)
Supplement: Supplementary file 1 [file cancers-11-00981-s001.pdf]

Supplementary Material: Neoadjuvant Treatment in Locally Advanced Pancreatic Cancer (LAPC) Patients with FOLFIRINOX or Gemcitabine NabPaclitaxel: A Single-Center Experience and a Literature Review

Fabiana Napolitano, Luigi Formisano, Alessandro Giardino, Roberto Girelli, Alberto Servetto, Antonio Santaniello, Francesca Foschini, Roberta Marciano, Eleonora Mozzillo, Anna Chiara Carratù, Priscilla Cascetta, Pietro De Placido, Sabino De Placido, and Roberto Bianco

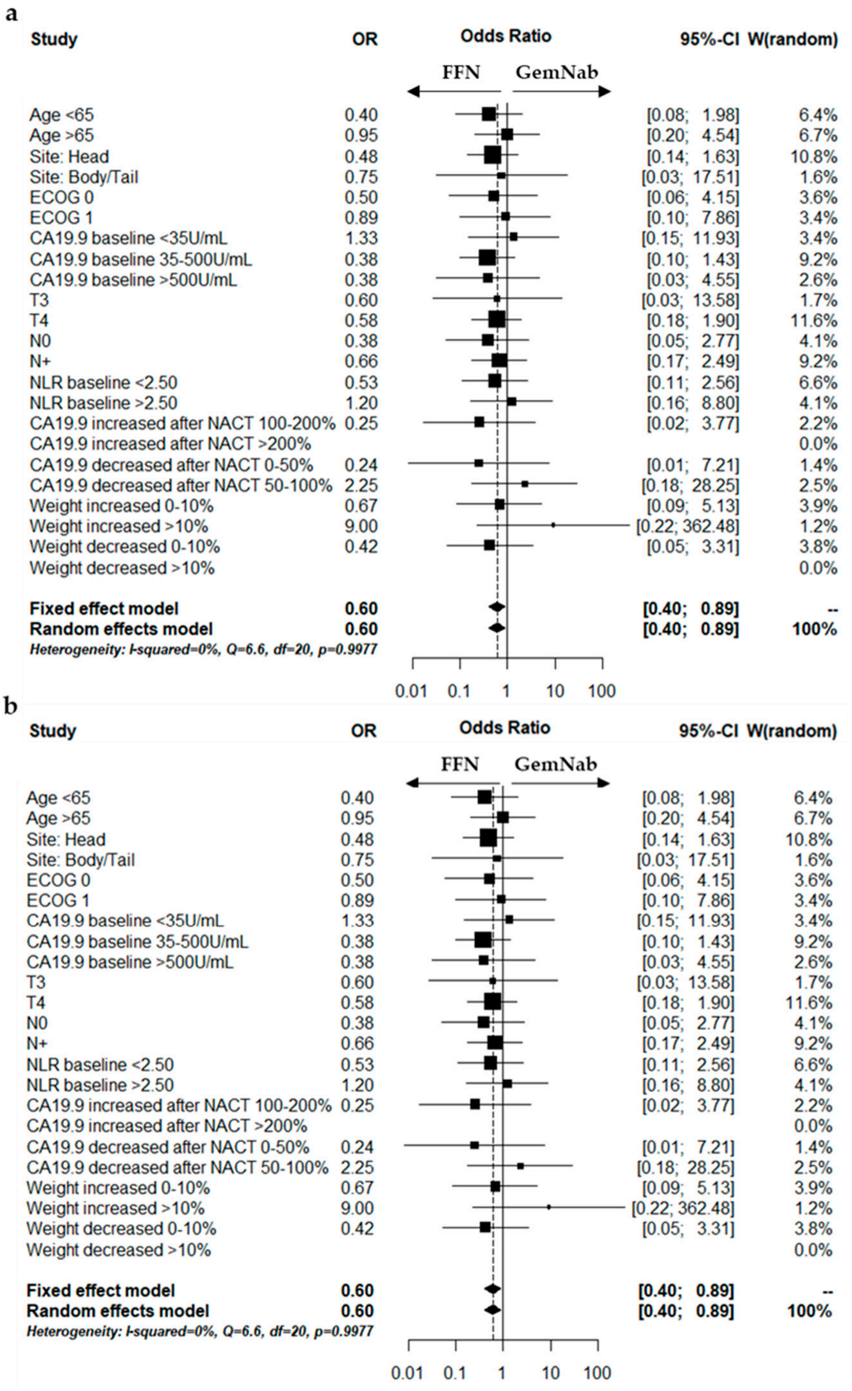

Figure S1. Forrest Plot. Evaluating the characteristics of the patients and the tumour, possibly influencing the (**a**) OS and (**b**) PFS.

Table S1. Active and/or ongoing clinical trials evaluating FOLFIRINOX and Gemcitabine NabPaclitaxel as neoadjuvant treatments.

Phase	Code	Title	Drugs Investigated	PRIMARY END-POINT
**Early Phase I**	NCT03600623	FOLFIRINOX or Gemcitabine-Nab Paclitaxel Followed by Stereotactic Body Radiotherapy for Locally Advanced Pancreatic Cancer	FOLFIRINOX or gemcitabine-nab paclitaxel (abraxane®) followed by Stereotactic Body Radiation Therapy (SBRT).	Safety
**Phase III**	NCT03690323	Pancreatic Locally Advanced Irresectable Cancer Ablation (PELICAN)	RFA plus standard palliative chemotherapy as compared to palliative chemotherapy alone in patients with LAPC after 2 months of induction chemotherapy.	OS
**Phase I**	NCT02873598	A Dose Escalation Trial of SBRT After Induction Chemotherapy for Locally Advanced Pancreatic Cancer	safety of stereotactic body radiotherapy (SBRT) delivered in 3 fractions for patients with locally advanced pancreatic cancer (LAPC) who have received induction chemotherapy (FOLFIRINOX or gemcitabine and nab-paclitaxel).	Safety
**Phase I/II**	NCT03641183	Neoadjuvant Therapy Followed by Stereotactic Body Radiotherapy (SBRT) for Locally Advanced Pancreatic Cancer	FOLFIRINOX or gemcitabine-nab paclitaxel (abraxane) followed by Stereotactic Body Radiation Therapy (SBRT).	Safety
**Phase II**	NCT02125136	Trial to Investigate Intensified Neoadjuvant Chemotherapy in Locally Advanced Pancreatic Cancer (NEOLAP)	FOLFIRINOX or gemcitabine-nab paclitaxel (abraxane)	Conversion Rate

© 2019 by the authors. Licensee MDPI, Basel, Switzerland. This article is an open access article distributed under the terms and conditions of the Creative Commons Attribution (CC BY) license (http://creativecommons.org/licenses/by/4.0/).
